# Recent advances in mucopolysaccharidosis IVA treatment

**DOI:** 10.1186/s13023-025-04028-0

**Published:** 2025-10-14

**Authors:** Andrés Felipe Leal, Harry Pachajoa

**Affiliations:** 1 Centro de Investigaciones en Anomalías Congénitas y Enfermedades Raras, Universidad Icesi, Cali, 760031, Colombia; 2 Centro de Investigaciones Clínicas, Fundación Valle de Lili, Cali, 760001 Colombia; 3 Departamento de Genética Clínica, Fundación Valle de Lili, Cali, 760001 Colombia; 4 Institute for the Study of Inborn Errors of Metabolism, Faculty of Science, Pontificia Universidad Javeriana, Bogotá, 110231 Colombia

**Keywords:** Adeno-associated virus, Antisense oligonucleotides, CRISPR/Cas9, ERT, GALNS, Lentivirus, MPS IVA

## Abstract

Mucopolysaccharidosis IVA (MPS IVA, Morquio A syndrome) is a rare lysosomal storage disorder caused by mutations in the GALNS gene, resulting in N-acetylgalactosamine-6-sulfatase (GALNS) deficiency and accumulation of keratan sulfate and chondroitin-6-sulfate. MPS IVA primarily affects the skeletal system, leading to progressive dysplasia and multi-organ involvement. Although enzyme replacement therapy (ERT) with elosulfase alfa is currently the only approved treatment, its clinical benefit on bone pathology is limited due to rapid clearance and poor penetration into avascular cartilage. Strategies to enhance enzyme stability and targeting, such as PEGylated hydrogels and extracellular vesicles, have shown promise in enhancing the biodistribution and stability of GALNS, while pharmacological chaperones, including ezetimibe, pranlukast, and bromocriptine, seem to stabilize GALNS in vitro. Promisingly, gene therapy (GT) has demonstrated significant preclinical progress using adeno-associated virus (AAV), lentiviral (LV), and CRISPR/Cas9 platforms. For instance, AAV vectors employing bone-targeting peptides and tandem promoters improve skeletal manifestations in murine and rat models. Similarly, recent ex vivo LV-based GT studies have opened new avenues in the treatment of MPS IVA. Furthermore, CRISPR/nCas9-based strategies targeting safe harbor loci have successfully restored GALNS activity in MPS IVA fibroblasts and mouse models supporting the notion that gene editing may represent a potential therapeutic approach. On the other hand, antisense-based therapies using modified U7 small nuclear RNAs and circular RNAs offer novel approaches to correct pseudoexon activation from deep-intronic variants while expanding the therapeutic alternatives in MPS IVA. Importantly, recent evidence revealed that mitochondrial dysfunction in chondrocytes may contribute to the pathology of MPS IVA, uncovering new targets beyond GALNS enzyme activity recovery. This review highlights recent advances in the treatment of MPS IVA and discusses new directions to improve outcomes in MPS IVA treatment.

## Introduction

Mucopolysaccharidosis IVA (MPS IVA; OMIM #253000), also known as Morquio A syndrome, is caused by mutations in the *GALNS* gene [1, 2]. *GALNS* is located on chromosome 16q24.3 and encodes the lysosomal enzyme N-acetylgalactosamine-6-sulfatase (GALNS), responsible for the degradation of keratan sulfate (KS) and chondroitin-6-sulfate (C6S) [1]. The lysosomal accumulation of KS and C6S leads to skeletal dysplasia, as well as cardiac and respiratory complications in patients [2]. MPS IVA common symptoms include short stature, pectus carinatum, hypermobile joints, genu valgum, and odontoid hypoplasia, increasing the risk of spinal cord compression [2]. Respiratory, cardiac, and surgical complications are the most common causes of death in MPS IVA patients [3].

Globally, the incidence of MPS IVA is estimated to be 1 in 200,000 to 1 in 300,000 live births [4]. Notably, in Latin American countries such as Colombia, Brazil, and Mexico, the incidence ranges from 0.68 to 3.0 per 100,000 live births [5–9]. Over the last decade, disease-modifying therapies have reshaped the therapeutic landscape. Enzyme replacement therapy (ERT) with elosulfase alfa remains the only Food and Drug Administration (FDA)-approved pharmacological treatment [10]. Despite systemic delivery, ERT exhibits poor diffusion into cartilage and growth plate areas [11, 12]. Hematopoietic stem cell transplantation (HSCT) has been used in MPS IVA patients [2, 12–14]. Even though HSCT has been shown to increase GALNS activity, improve pulmonary function, and enhance bone mineral density [14], it is not recommended as a standard therapy for MPS IVA due to the risk of graft-versus-host disease (GVHD) [15, 16], its limited impact on height and skeletal dysplasia, and the lack of robust supporting evidence [12]. Pharmacological chaperones (PCs) are emerging treatments that can induce the stabilization of GALNS [17–19], but their effectiveness has not been tested preclinically.

Gene therapy (GT) is a promising strategy for treating MPS IVA. It has been evaluated using adeno-associated virus (AAV), which has shown encouraging outcomes in preclinical models, paving the way for the first GT clinical trial, which started in 2023 [20]. Lentiviral (LV)-based GT has recently shown favorable advances in ex vivo GT [21]. Moreover, CRISPR/Cas9-based genome editing (GE) is being investigated for the insertion of expression cassettes at safe harbors [22–25], offering the potential for a permanent cure with a single administration.

This review summarizes the latest advancements in therapies for treating MPS IVA and outlines future directions for the field.

## Enzyme replacement therapy (ERT)

**Fig. 1:**
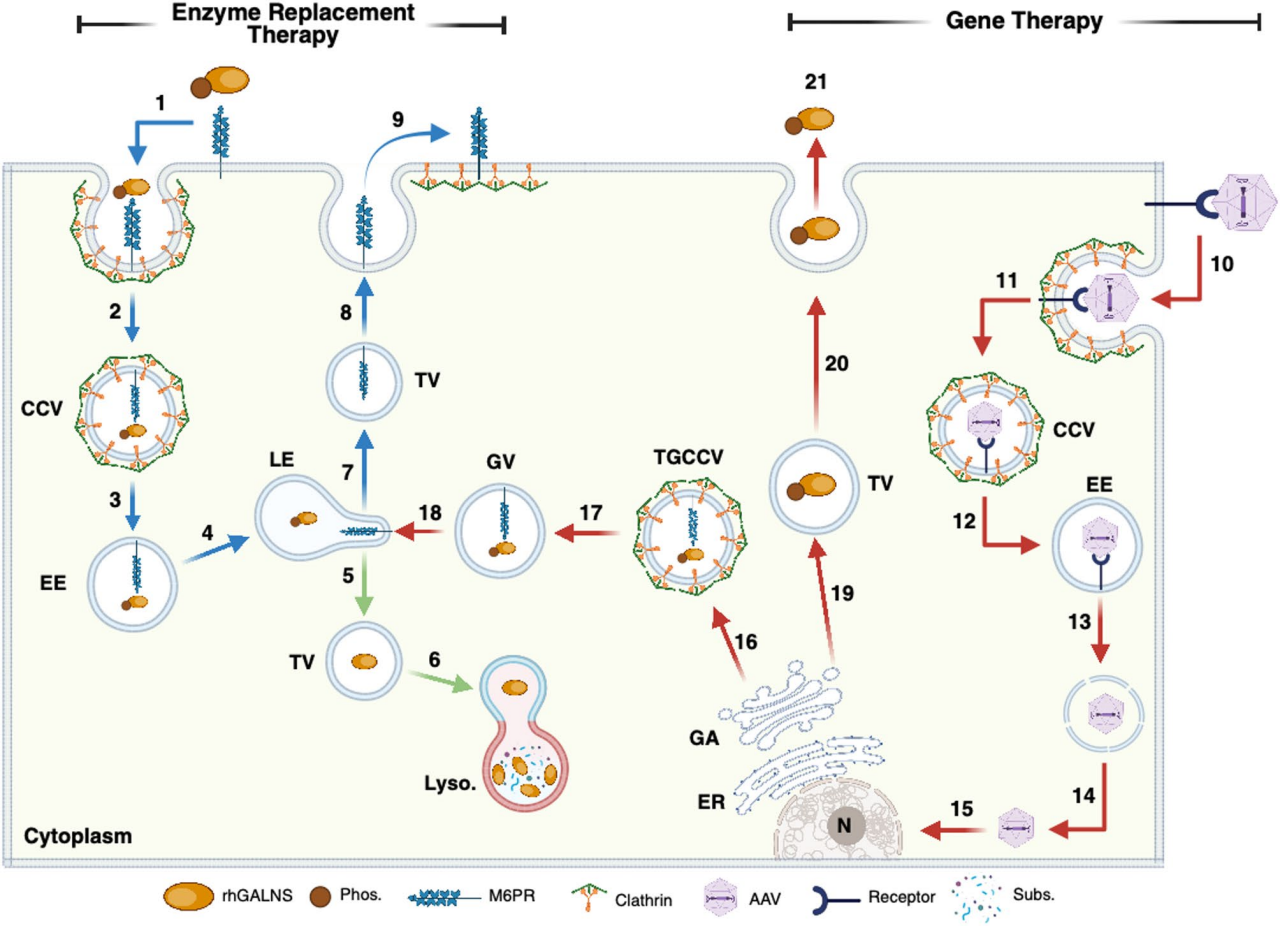
Enzyme supply strategies. Enzyme replacement therapy (ERT) and GT represent the primary alternatives explored to restore the GALNS enzyme in deficient MPS IVA cells. Initially, in ERT-based approaches, the rhGALNS is recognized by mannose 6-phosphate receptors (M6PR) at the plasma membrane (PM) (**1**), which triggers its clathrin-dependent endocytosis (**2**). Later, the rhGALNS enzyme is sorted into clathrin-coated vesicles (CCV) within cells through early (EE) (**3**) and late (LE) (**4**) endosomes. Upon dissociation of rhGALNS from M6PR, rhGALNS is transported to the lysosomes (Lyso.) (**5**), where it is expected to degrade C6S and KS (**6**). M6PR is recycled from LE (**7**) in the transport of vesicles (TV) (**8**) to the plasma membrane (**9**). On the other hand, GALNS restoration can also be achieved via GT. The carrier (i.e., adeno-associated virus (AAV)), transporting the GALNS expression cassette, is recognized by specific receptors at the PM (**10**), which leads to the carrier’s endocytosis (**11**). While EEs also traffic the carrier sorting (**12–13**), as observed in ERT approaches, for GT, it is required that the carrier can escape from the EE to reach the cytoplasm (**14**) and later the nucleus (N) (**15**). In the nucleus, the DNA is released, and the cell’s transcription machinery performs gene transcription. The resulting mRNA undergoes translation in the endoplasmic reticulum (ER) and later post-translational modification, including M6P modification, in the Golgi apparatus (GA). Resulting Trans-Golgi clathrin-coated vesicles (TGCCV) sort GALNS (**16**) within the intracellular trafficking to LE (**17–18**). Later sorting in lysosomes occurs as observed during ERT. Importantly, GT approaches aimed not only to supply GALNS enzyme in the targeted cells but also to promote GALNS release into the extracellular medium via TV (**19–20**). Released GALNS can then be taken up by neighboring cells in an M6PR-dependent mechanism (**21**), thus mediating a cross-correction mechanism. Blue, red, and green arrows represent ERT, GT, and common intracellular pathways, respectively. This figure was created with Biorender.com

Chinese hamster ovary (CHO) cell-produced recombinant human GALNS (rhGALNS) was approved as an ERT by the FDA in 2014 for the treatment of MPS IVA patients [10, 11]. ERT is based on the ability that mammalian cells have to uptake lysosomal enzymes via the mannose 6-phosphate receptor (M6PR). Upon its uptake, rhGALNS is sorted into the endolysosomal pathway, ultimately reaching the lysosome, where it is expected to hydrolyze sulfate groups from KS and C6S (Fig. 1).

ERT has demonstrated efficacy in improving the 6-minute walk test, the 3-minute stair climb test, and respiratory function when compared with placebo-treated MPS IVA patients [10, 11, 26]. Nevertheless, ERT has a limited impact on bone pathology, as it is unable to reach the avascular zones of the bone [26]. ERT is currently intravenously administered, and it requires pre-treatment with antihistamines with or without antipyretic, 20 to 60 min before infusion starts [27]. The development of side effects, including anaphylaxis, is a significant challenge when administering ERT. Desensitization protocols are necessary to reduce the risk of adverse reactions in patients [28–30] and to prevent ERT interruption, thereby maintaining the disease and patients’ quality of life as stable as possible [31].

A Phase 1 clinical trial (NCT04532047) is evaluating the prenatal ERT administration in pregnant women with genetic or enzymatic confirmation of MPS IVA, and the pregnancy is between 18 and 34 weeks. Although no findings have been reported yet (expected to be completed by 2031), *in utero* ERT could overcome the current limitation of ERT in reaching the bone, as the bone is still developing during early pregnancy periods. Indeed, secondary ossification centers become visible by the second trimester, while in the third trimester, the bones continue growing [32, 33]. Additionally, in utero, ERT is expected to enhance the postnatal response to ERT, as fetal ERT administration may promote immune tolerance [34–36].

Several expression systems have been used to express rhGALNS [37–39]. A recent study conducted by Pimentel et al.. (2024) aimed to produce rhGALNS in a glyco-engineered *Escherichia coli* (*E. coli*) strain able to add trimannosyl core N-glycan (Man_3_GlcNAc_2_), an eukaryotic-like N-glycan, to rhGALNS during its heterologous expression, thus enabling the enzyme to be uptaken by mammalian cells through M6PR [39]. The resulting N-glycosylated rhGALNS (rhGALNSoptGly) exhibited enhanced pH stability and improved kinetic parameters compared to non-glycosylated versions. Most importantly, rhGALNSoptGly was successfully internalized by MPS IVA fibroblasts and demonstrated a 2-4-fold increase in intracellular GALNS enzyme activity after 24 h of incubation compared to wild-type (WT) fibroblasts [39]. Consistently, the authors reported a significant decrease in KS levels upon rhGALNSoptGly treatment in MPS IVA fibroblasts. Interestingly, 48 h post-incubation, GALNS enzyme activity and KS returned to the levels observed in untreated MPS IVA fibroblasts, suggesting the rapid clearance of rhGALNSoptGly [39]. Preclinical assessment remains necessary to demonstrate the therapeutic efficacy of rhGALNSoptGly and to evaluate the effect of the added N-glycosylation on immune response activation.

CHO-derived rhGALNS requires weekly administration, making it an expensive strategy (∼380,000 USD per year per patient) [40, 41]. This is due, in part, to the short half-life of GALNS and its low stability at 37 °C [42]. To overcome these drawbacks, recent studies have attempted to develop Poly(ethylene glycol) (PEG)-based hydrogels to enhance GALNS stability while facilitating sustained GALNS release [43–46]. In this regard, Flanagan et al.. (2023) developed an eight-arm polyethylene glycol-acrylate macromer PEG hydrogel that led to increased degradation time, preserving rhGALNS enzyme activity for 28 days, which resulted in higher preservation than the 7 days observed with GALNS stabilization buffer (25 mM sodium acetate, 1 mM β-glycerophosphate, pH 5.5) [45]. The rhGALNS structure remained unaffected upon PEGylation reactions, suggesting that the encapsulation has no negative impact on the rhGALNS conformation. In C57BL/6 albino mice, subcutaneously injected hydrogel-encapsulated rhGALNS remained detectable for up to 20 days, compared to only 7 days for the enzyme delivered in buffer [45], supporting that PEG-based hydrogels preserve and release rhGALNS longer than standard ERT [42]. Although PEG-encapsulated rhGALNS still needs to be evaluated in MPS IVA mouse models to establish its therapeutic efficacy, this is a promising finding that could potentially shorten ERT dosing frequency in MPS IVA patients, thereby improving their quality of life, as fewer visits to infusion centers would be required.

Increasing evidence supports that extracellular vesicles (EVs) can be used as delivery platforms for ERT in several LSD [47–51], acting as molecular trojan horses [52]. In fact, it was recently demonstrated that native mesenchymal stem cell-derived EVs (MSC-EVs) transport bioactive GALNS enzyme [51]. The characterization of MSC-EVs has demonstrated heterogeneous populations characterized by exosomes (CD53/CD151) and microvesicles (PECAM1/CD14) [51]. As shown in various mammalian cells [47–51], MSCs are also suitable for inducing GALNS overexpression [51], indicating that they can be designed to transport large amounts of the GALNS enzyme. Therefore, the molecular Trojan horse activity of MSC-EVs, along with their regenerative and immunomodulatory properties [53, 54], could provide a novel alternative for treating MPS IVA.

## Pharmacological chaperones (PCs)

PCs are small molecules designed to stabilize misfolded proteins by aiding their proper conformational structure within the endoplasmic reticulum [55, 56]. In this regard, ezetimibe [18], pranlukast [18], and bromocriptine [17] have been demonstrated to have chaperone activity in several mutations affecting the *GALNS* gene (Table 1). Although these PCs have the potential to increase residual GALNS enzyme activity in MPS IVA fibroblasts while decreasing lysosomal mass and partially restoring autophagy flux [17, 18], the in vivo evaluation of PCs is lacking, which limits their clinical translation.

**Table 1:** Effect of Pharmacological chaperones on RhGALNS enzyme activity

GALNS enzyme activity increase (Fold-Change)							
		Ezetimibe	Pranlukast	Bromocriptine		Comp. 1	Comp. 36
Source/ID	Mutation	0.001	0.001	0.1	10	500	30
*GM00593	p.R386C, p.F285del	2.5	UC	UC	1.5	-	-
*GM00958	p.A393S	2.5	~ 1.3	UC	UC	-	-
*GM01259	p.R94C, p.A393S	1.4	UC	NT	NT	-	-
*GM01361	p.R61W, p.W405 T406del	1.6	~ 1.5	1.5	Dec.	-	-
**E. coli	WT rhGALNS	I: UC	I: UC	NT	NT	-	-
		E: 1.3	E: 1.7			-	-
*K. phaffii*	WT rhGALNS	I: UC	I: UC	NT	NT	-	-
		E: 5.0	E: ~2.3			-	-
HEK293	WT rhGALNS	I: UC	I: 1.3	UC	1.48	-	-
		E:1.6	E:1.8			-	-
CHO	WT rhGALNS	-	-	-	-	2.0	2.4

*Corresponds to human MPS IVA fibroblasts obtained from Coriell Institute for Medical Research. **E. coli BL21 (DE3). *Comp* Compounds 1 and 36 refer to two pyrrolidine-based nonavalent dendrimers, *UC* Unchanged, *I* Intracellular, *E* Extracellular, *NT* Not tested, *Dec* Decreased rhGALNS enzyme activity, 0.001, 0.1, 10, 500, and 30 correspond to PCs’ concentration expressed in µM. Data were extracted from [17–19, 56].

The production of rhGALNS has been attempted in several expression systems, including mammalian cells [42], yeast [57], and bacteria [39]. Nevertheless, low rhGALNS stability remains a critical concern [42]. Ezetimibe and pranlukast were shown to increase extracellular GALNS enzyme activity in HEK293, *E. coli*, and *Komagataella phaffii* [18], supporting their chaperone activity on unaffected GALNS enzymes. Similar findings were also observed with Bromocriptine in rhGALNS produced in HEK293 cells [17]. Recently, Davighi et al.. (2025) reported a library of multivalent glycomimetics that could act as PCs for stabilizing rhGALNS [19]. Among several candidates, the biological evaluation identified two pyrrolidine-based nonavalent dendrimers, designated as 1 and 36, potentially having chaperone activity. In this sense, the potential stabilizing effect mediated by these compounds was assessed in thermal denaturation experiments at 48 °C for 40 and 60 min [19]. During these experiments, the authors observed that compound 1 protected rhGALNS enzyme activity (a 2-fold increase compared to untreated rhGALNS) at a concentration of 500 µM across both time points evaluated.

In contrast, when assessing compound 36, rhGALNS enzyme activity (1.3-fold increase compared to untreated rhGALNS) was preserved at a concentration of 30 µM for 40 min [19]. Conversely, rhGALNS enzyme activity was significantly higher (2.4-fold increased) after 60 min than that observed with untreated rhGALNS [19]. These findings further support the previous chaperone activity observed with ezetimibe, pranlukast, and bromocriptine for rhGALNS, opening a new avenue for using PCs in conjunction with standard ERT. This novel strategy could increase the half-life of rhGALNS, thereby decreasing the frequency of ERT infusions. Further preclinical studies are needed to confirm this premise.

## Gene therapy (GT)

GT is a promising approach for treating monogenic diseases, such as MPS IVA. Meaningful progress using viral and non-viral delivery systems has enabled approaches aimed at inducing long-term expression of GALNS. In this regard, AAV-, LV-, and CRISPR/Cas9-based GT have been evaluated for the treatment of MPS IVA using in vitro, in vivo, and ex vivo approaches. Table 2 summarizes the primary outcomes of preclinical studies for MPS IVA.

**Table 2:** Main findings in preclinical studies in MPS IVA

Appr	Model	Vector	*Vector dose	Outcome & dosing conclusion	Reference
IV	MKC MTOL	AAV8-TBG-hGALNS AAV8-CAG-hGALNS, with/without D8.	5 × 10¹³	Treatment increased GALNS activity, normalized KS levels, and improved skeletal/cardiovascular features. MTOL mice responded better than MKC mice.	[58]
IV	MKC		TBG: 2 × 10^14^ CAG: 5 × 10^13^	CAG vectors are more efficient than TBG, even at lower doses. CAG better-improved bone lesions in the presence or absence of D8.	[59]
IV	MKC		TBG: 5 × 10^13^ 2 × 10^14^ CAG: 5 × 10^13^	Females had higher antibodies and lower efficacy. Males had a better response. Sex significantly influences GT outcome.	[60]
IV	MKC2	AAV8-CAG-hCNP	3.5 × 10^13^	CNP induced chondrocyte proliferation and bone growth.	[61]
IV	MKC2	AAV9-CAG-hGALNS AAV8CAG-hCNP	GALNS: 4 × 10¹³ CNP: 4 × 10⁹, 1 × 10¹², and 4 × 10¹³	Combination therapy improved bone growth and enzyme activity more than GALNS alone. Higher CNP doses had a greater effect, although they induced overgrowing and movement difficulty in animals.	[62]
IV	MKC	AAV8-TBG-hGALNS AAV8-LSPX-hGALNS AAV8/9-CAG-hGALNS AAV8/9-LMTP-hGALNS AAV8/9-LBTP-hGALNS	5 × 10^13^	Liver-specific promoters produced high GALNS enzyme activity and KS normalization but failed to significantly improve bone pathology. CAG promoter offered broader tissue distribution, moderate GALNS activity in muscle and bone, and led to partial bone correction. Tandem promoters, especially AAV9-LMTP, outperformed all others, achieving the greatest amelioration of pathological features.	[63]
IV	R388C	AAV9-CAG-rGALNS	6.6 × 10^13^	Long-term (1-year) increase in GALNS, reduced KS levels, and full skeletal dysplasia correction.	[64]
EV	MKC2	LV-CBh-hGALNS LV-COL2A1-hGALNS	MOI 60–0.5–0.9 × 10⁶ HSCs/mouse	CBh promoter led to higher GALNS enzyme activity in plasma, liver, and spleen but less skeletal correction. COL2A1 promoter enabled better heart and skeletal outcomes despite lower enzyme levels.	[21]
IV	MTOL	IONPs-nCas9 plasmid- CAG-hGALNS plasmid	2.5 µg IONPs + with 3.8 µg pDNA	Increased GALNS activity, reduced KS, partial bone recovery, no liver or kidney toxicity, or iron homeostasis alteration. No off-target or anti-nCas9 Abs.	[23]

*Dose in AAV vectors is expressed as GC/kg. *Appr* Approach, *CAG* Cytomegalovirus Early Enhancer/Chicken β-Actin Promoter, *CBh* Chicken β-Actin Hybrid Promoter fused to CMV enhancer, *D8* is a bone-targeting peptide, *EV Ex vivo*, *hGALNS* Human GALNS, *IONPs* Iron Oxide Nanoparticles, *IV In vivo*, *LBTP* Liver-Bone Tandem Promoter, *LMTP* Liver-Muscle Tandem Promoter, *LSPX* Liver-Specific Promoter based on the human alpha1-antitrypsin (hAAT), *MKC* MPS IVA knockout mouse (KO), *MKC2* The MPS IVA KO mouse was generated with a larger deletion site than MKC, *MOI* Multiplicity of Infection, *MTOL* MPS IVA knockout mouse tolerant to hGALNS, *TBG* Thyroxine-Binding Globulin Promoter, *rGALNS* Rat GALNS, *R388C* Corresponds to the rat model carrying the Arg388Cys mutation on the GALNS gene.

### AAV-based GT

Adeno-associated virus (AAV)-based GT utilizes the natural ability of AAV to infect mammalian cells through receptor-mediated endocytosis [65, 66]. Upon endocytosis, viral particles reach the nucleus via endosomal escape, allowing the virus to enter the cytoplasm and subsequently the nucleus (Fig. 1). The AAV genome remains as an episome in the nucleus, where gene transcription occurs, mediated by the cell’s transcription machinery [65]. Various AAV serotypes have been identified, differing in their cell tropism, which makes them a valuable tool for GT in targeting specific tissues. Significant limitations of AAV include preexisting anti-AAV antibodies, vector dilution, limited packing capacity (4.7 kb), and potential off-target effects [67, 68]. Despite these challenges, AAV has shown promising outcomes in preclinical assessments using MPS IVA mouse models [21–25, 58, 59, 61–63, 69] and rat models [64].

Initial studies conducted by Sawamoto et al. (2020) evaluated the efficacy of AAV8 in delivering hGALNS and correcting systemic and bone manifestations in 4-week-old MPS IVA mice [58]. AAV8 was engineered to express or not the bone-targeting aspartic acid octapeptide (D8) upstream of the hGALNS cDNA sequence driven by the liver-specific promoter TGB [58]. D8 increases the vector affinity by hydroxyapatite [70]. This study demonstrated that AAV8-mediated GT results in supraphysiological levels of the hGALNS enzyme in several tissues, leading to a decrease in KS in plasma, liver, and lung. Most importantly, AAV8-GT decreases chondrocyte vacuolization in the articular cartilage and plate growth region, thus suggesting that bone pathology is ameliorated while nearly correcting heart pathology [58]. Although plasma hGALNS levels were significantly higher when mice were treated with D8-expressing hGALNS, it did not lead to a superior outcome in bone pathology recovery when compared to unmodified hGALNS [58]. Later experiments performed by Herreño-Pachón et al.. (2024) found comparable efficacy in MPS IVA mice, supporting the notion that AAV8-mediated GT is a potential strategy for treating MPS IVA [59].

The immune response raises a crucial concern in AAV-based GTs. To assess the influence of sex on the presence of anti-hGALNS neutralizing Abs and GT efficacy, Piechnik et al.. (2022) treated young male and female MPS IVA mice with AAV8 vectors carrying TGB-GALNS cDNA under the control of TGB and the ubiquitous promoter CAG [60]. In this study, the authors found that female mice elicited up to a 4.6-fold higher level of anti-AAV neutralizing Abs compared to male mice, along with a lower hGALNS enzyme activity level (6.8-fold lower) resulting from a decline in hGALNS levels over time [60]. Consistently, bone and cardiac pathology were notably improved in male mice compared to female mice [60]. Similar findings were reported regardless of the promoter driving hGALNS expression. These findings outline the importance of evaluating the sex influence in GT strategies.

A recent study published by Rintz et al.. (2023) demonstrates the potential use of AAV8 for expressing the C-type natriuretic peptide (CNP) to induce bone growth in MPS IVA mice [61]. CNP is a small bone-penetrating molecule that stimulates bone growth by interacting with the natriuretic peptide receptor B (NPR-B) in proliferative and pre-hypertrophic chondrocytes [61]. It has been evaluated for treating certain skeletal dysplasias, such as achondroplasia [71]. In the study conducted by Rintz et al.. MPS IVA mice treated with AAV8-CNP exhibited a significant increase in body length and weight compared to untreated counterparts, with notable differences observed from week 5 post-treatment onward. Significantly, AAV8-CNP treatment effectively decreases KS levels in plasma while normalizing them in bone [61]. Likewise, chondrocytes at the growth plate were less vacuolated and more organized in a columnar structure than in untreated MPS IVA mice. Bone architecture, evaluated using micro-CT, consistently revealed increased trabecular volume, decreased trabecular thickness, and an increase in total cortical medullary area [61].

Although the findings reported by Rintz et al.. support that CNP could become a potential alternative for inducing bone growth, a study published by Ago et al.. (2025) showed that N-terminal pro-C-type natriuretic peptide (NT-proCNP) is found elevated in MPS IVA patients [72], raising new concerns about the safety of the CNP overexpression through AAV-based vectors, as CNP regulates vital processes such as vascular homeostasis and blood pressure [73, 74]. In this regard, later studies conducted by Rintz et al.. (2024) showed that, despite the significant improvement in bone pathology, high AAV8-CNP doses (4 × 10^3^ GC/Kg) result in excessive bone growth in MPS IVA mice, leading to difficulty in movement in animals [62], further suggesting that CNP dosing is critical to avoid deleterious outcomes. By decreasing the AAV8-CNP dose (1 × 10^12^ GC/Kg), the authors found that the combination therapy, composed of AAV9-hGALNS and AAV8-CNP, led to a significant increase in hGALNS enzyme activity and a decrease in KS in several tissues, along with amelioration of bone pathology [62]. Co-expression of GALNS and CNP in AAV9 as a single expression cassette dramatically decreases GALNS enzyme activity detection in some tissues (i.e., plasma, bone), thereby barely improving bone pathology in MPS IVA mice [62]. Globally, this study outlines the potential of combination therapies using vectors with different tropisms to express hGALNS along with key bone modulators such as CNP.

Most recently, a study conducted by Khan et al.. (2025) compared the efficacy of AAV-based GT using several promoters, including CAG, TBG, LMTP, and LBTP, in ameliorating the pathological findings in an MPS IVA [63]. In this study, the authors found that CAG (∼1100% WT levels) and TBG (∼1500% WT levels) yielded the highest GALNS enzyme activity in plasma compared to LMTP (∼300% WT levels) and LBTP (∼800% WT levels) when using AAV8 vectors after 8 weeks post-treatment [63]. When using AAV9, GALNS enzyme activity in plasma was similar in LMTP (∼1200% WT levels) and LBTP (∼900% WT levels) to that observed with AAV8-TBG and AAV8-CAG. In the femur, only AAV8-CAG and AAV9-LMTP induced supraphysiological levels of the GALNS enzyme; nevertheless, KS was normalized to WT in all vectors and promoters tested, regardless of the achieved GALNS enzyme activity [63]. AAV9-LMTP led to higher GALNS enzyme activity in muscle and bone compared to other promoters. Consistently, a significant decrease in KS levels in the muscle was observed [63]. This study highlights the therapeutic efficacy of using different serotypes and promoters.

Although most of the current data in GT have been generated in MPS IVA mouse models, the lack of a skeletal dysplasia phenotype is a significant limitation [1]. In 2021, Bertolin et al.. developed an MPS IVA rat model by inducing a point mutation (Arg388Cys) at the rat *GALNS* gene, which corresponds to the Arg386Cys mutation in humans [64]. The novel MPS IVA rat model recapitulates the skeletal and non-skeletal alterations expected in humans, thus providing a more accurate MPS IVA animal model. Most importantly, AAV9-based GT in MPS IVA rats resulted in vector transduction in bone, cartilage, and peripheral tissues. Consistently, the authors reported that AAV9-GALNS administration corrected bone, cardiac, and tracheal alterations in 4-week-old MPS IVA-treated rats [64]. Although the findings in this study showed the potential of AAV9 in reaching and transducing avascular regions in the bone, the authors used a rat GALNS cDNA, which lacked evidence of these promising findings when using hGALNS cDNA. Rat-derived anti-hGALNS neutralizing Abs could decrease the therapeutic response as observed in mouse models [63]. Humanized rats (i.e., expressing truncated hGALNS) could offer a novel approach to testing the therapeutic efficacy of AAV9-hGALNS in this innovative MPS IVA animal model.

### LV-based GT

 Lentiviruses, a subclass of retroviruses, have gained particular interest due to their ability to integrate into the host genome and transduce both dividing and non-dividing cells [75]. Originally derived from the human immunodeficiency virus (HIV-1), lentiviral vectors (LVs) have been extensively engineered for enhanced biosafety and transduction efficiency, leading to their adoption in various clinical trials, including those for primary immunodeficiencies and hemoglobinopathies [76, 77]. LV-mediated transgene delivery offers key advantages over AAV, as it has a larger packaging capacity and a lower immunogenic profile [75, 77]. Nevertheless, insertional mutagenesis, random integration, and epigenetic silencing remain the most significant challenges [78]. In MPS IVA, several in vitro and ex vivo approaches have evaluated the efficacy of LV-mediated GT.

Initial studies performed by Puentes-Tellez et al.. (2021) demonstrated the suitability of HIV-derived LVs for transducing MPS IVA fibroblasts [79]. Upon transduction with LVs carrying hGALNS cDNA alone or in combination with hSUMF1 cDNA, a significant increase in the GALNS enzyme activity, a decrease in the lysosomal mass, and autophagy flux recovery were noticed [79]. Mutation-causing GALNS deficiency influenced the successful recovery of biomarkers in MPS IVA fibroblasts [79], suggesting that some mutations may lead to differential responses in LV-based GT approaches.

Most recent studies by Celik et al.. (2024) evaluated, for the first time, the use of LV in an ex vivo approach using mouse HSCs [21]. In this study, the authors aimed to test the efficacy of LVs carrying hGALNS expression cassettes under the control of ubiquitous (CBh) or cartilage-specific (COL2A1) promoters. LVs were used to transduce murine HSCs, which were then transplanted into busulfan-treated MPS IVA mice [21]. Interestingly, both vectors led to supraphysiological hGALNS activity in white blood cells (WBC) from treated MPS IVA mice, suggesting that LV-mediated HSC transduction does not affect the in vivo differentiation of HSC into mature blood circulating cells [21]. Besides, LV-CBh-hGALNS-treated mice showed detectable levels of the GALNS enzyme in the tibia, while LV-COL2A1-hGALNS-treated mice did not. Interestingly, in LV-COL2A1-hGALNS-treated mice the KS levels were normalized to WT levels, while LV-CBh-hGALNS-treated mice showed a non-significant KS decreased in bone (humerus) [21]. Similarly, LV-COL2A1-hGALNS treatment completely rescued the heart pathology, whereas LV-CBh-hGALNS failed to recover the cell vacuolization in the heart to the same extent as LV-COL2A1-hGALNS, although a slight improvement was observed [21]. LV-COL2A1-hGALNS and LV-CBh-hGALNS partially corrected bone pathology. Additionally, a non-significant improvement in bone microarchitecture was observed in MPS IVA mice treated with COL2A1-hGALNS [21]. Although ex vivo LV-based GT still needs to be optimized to achieve complete bone correction, including bone microarchitecture, this pioneering study offers proof-of-concept for investigating ex vivo GT in the treatment of MPS IVA.

###  CRISPR/Cas9-based GT

**Fig. 2:**
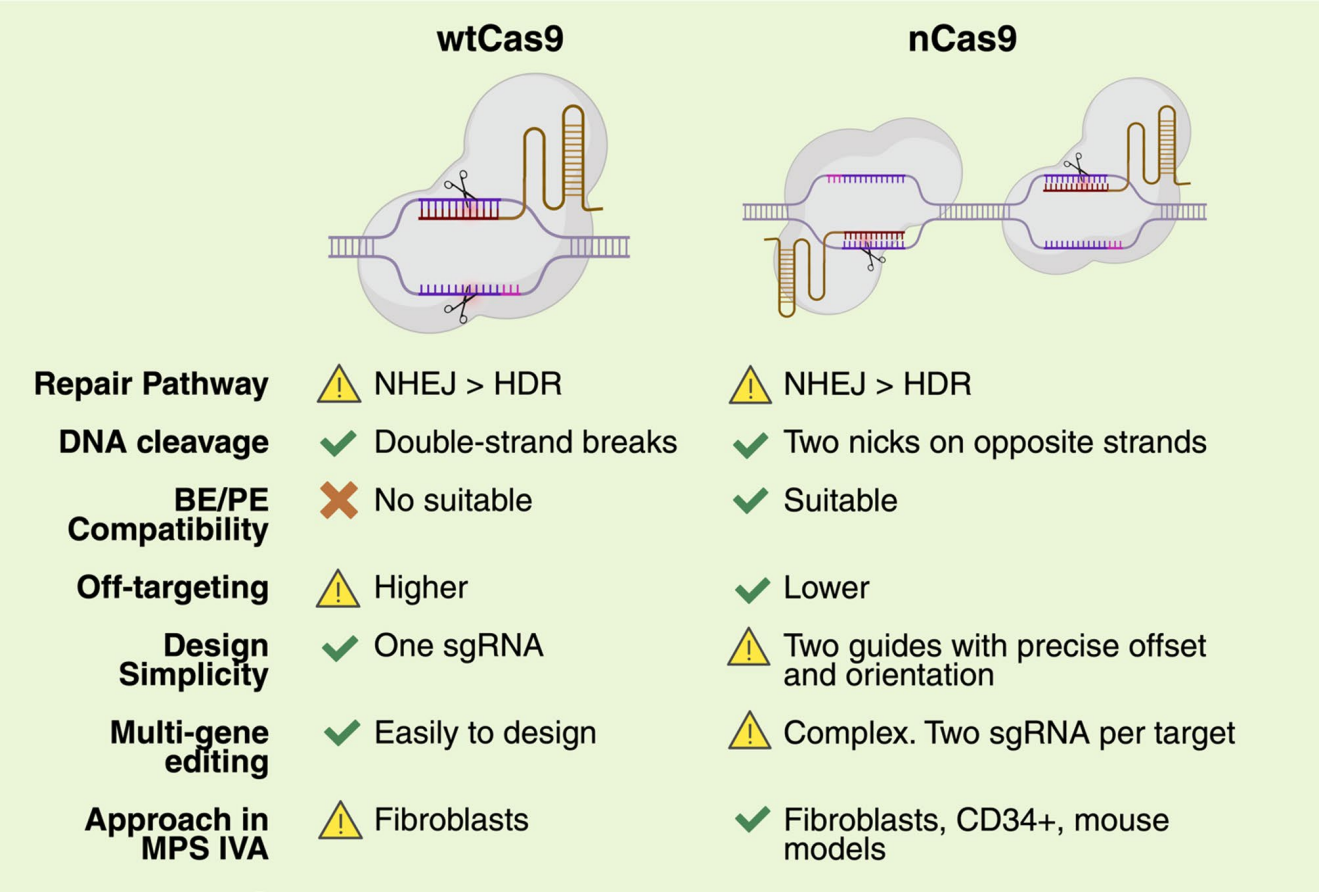
CRISPR/Cas9 alternatives evaluated MPS IVA models. Key features of Cas9 wild-type (wtCas9) and nickase (nCas9), including preferred DNA repair pathways, base/prime editing compatibility, off-target potential, design complexity, and relevance for multi-gene editing. Applications in MPS IVA are highlighted, showing the broader utility of nCas9 in both fibroblasts and hematopoietic cells, *HDR* Homologous repair, *NHEJ *Non-homologous end-joining. sgRNA. Single guide RNA. This figure was created with Biorender.com

 The CRISPR/Cas9 system enables gene modification, including gene knockout, knocking and base editing [80, 81]. Unlike traditional GT using AAV and LV, the CRISPR/Cas9 system enables the direct correction of pathogenic mutations at their native loci, potentially restoring normal gene function with long-term durability [80]. Most promising, the CRISPR/Cas9 system allows the insertion of long-expression cassettes at safe harbors [82, 83], resulting in the recovery of gene expression regardless of the disease-causing mutation. This is particularly interesting for MPS IVA, as more than 350 mutations have been identified in the GALNS gene [1]. The CRISPR/Cas9 system, primarily utilizing Cas9 nickase (nCas9), has been tested in MPS IVA fibroblasts [24, 25, 69] and CD34 + cells [22], as well as in MPS IVA mouse models [23], using both non-viral vectors (NVVs) and viral vectors (VVs). Figure 2 shows a comparison between wtCas9 and nCas9, which have been evaluated in models of MPS IVA.

In vitro, Leal and Alméciga (2022) demonstrated, for the first time, the efficacy of a CRISPR/Cas9 system to insert an expression cassette carrying hGALNS cDNA at the AAVS1 locus [24]. The AAVS1 locus is a safe insertion site that enables the knock-in of foreign DNA without causing cell disturbances [84]. In the studies conducted by Leal & Alméciga (2022), the authors employed a Cas9 variant termed Cas9 nickase (nCas9), as it has been shown to decrease the potential for off-targeting while preserving its ability to induce high knock-in efficiencies [85, 86]. Consistently, DSB-mediated nCas9 was as high as 37%, while unwanted Cas9 cutting was not observed in 10 in silico-predicted off-target sequences [24]. Interestingly, 30 days post-treatment, the authors found a recovery of up to 40% in GALNS enzyme activity, which was sufficient to normalize lysosomal mass, mitochondrial-dependent oxidative stress, and global GAG accumulation [24], providing novel evidence of the CRISPR/nCas9 efficacy in treating MPS IVA. Recent studies published by Suarez et al.. (2025) further support the suitability of the CRISPR/Cas9 system for recovering the MPS IVA fibroblast phenotype [69].

A later study, conducted by Leal et al.. (2022), aimed to evaluate the transport and delivery of the CRISPR/Cas9 system using iron oxide-based nanoparticles (IONPs) [25]. In this study, the authors found that IONPs yield comparable phenotype recovery in human MPS IVA fibroblasts carrying different GALNS-affecting mutations, compared to the phenotype recovery observed when using lipofectamine [25], supporting the notion that NVVs could be a novel alternative to VVs for delivering the CRISPR/Cas9 system.

Moving towards a preclinical assessment, the CRISPR/nCas9 system was also administered to MPS IVA mice using IONPs [23]. Initial experiments demonstrated that IONPs accumulated primarily in the liver, followed by the lungs, arms, legs, spleen, and kidneys, suggesting a widespread biodistribution [23]. Liver, renal, and iron homeostasis showed no toxicity related to IONP administration, indicating the dose used (2.5 µg IONPs carrying 3.8 µg CRISPR/Cas9 plasmids) was safe for animals [23]. The CRISPR/Cas9 system led to a 28% recovery of GALNS enzyme activity in the plasma of treated MPS IVA mice. Significant GALNS enzyme activity increase was also reported in the liver, muscle, spleen, heart, kidneys, and lungs. Conversely, a slight, non-significant increase in GALNS was observed in the tibia and trachea [23]. Biochemical correction was observed in plasma, liver, and humerus, suggesting that non-supraphysiological GALNS levels can mediate phenotype correction as observed in classical AAV- and LV-mediated GT [58]. Despite the significant decrease in KS in the bone, the vacuolization of the chondrocytes was only partially corrected [23], further highlighting the need for strategies targeting the avascular zones of the bone that remain unaddressed.

Although in vivo assessment of the CRISPR/Cas9 has shown encouraging results, the preexisting immune response against Cas9 proteins [87] could limit its therapeutic efficacy. A study conducted by Herreño-Pachón et al. (2025) aimed to evaluate the GE efficacy of the CRISPR/nCas9 system for modifying HSCs [22]. CRISPR/nCas9-based knock-in did not affect HSCs’ properties and led to supraphysiological GALNS expression [22]. The authors also observed an increase in intracellular GALNS in MPS IVA cells upon co-culture with edited HSCs. Normalization of classical MPS IVA biomarkers was observed in MPS IVA cells [22], suggesting cross-correction. The transplantation of CRISPR/nCas9-edited CD34 + cells into MPS IVA mouse models still needs to be conducted.

## Emerging alternatives

### Small molecules-CNP

As previously discussed, AAV8-based GT has been shown to promote bone growth in MPS IVA mice [61, 62]. Currently, a Phase I/II open-label study is evaluating the safety and tolerability of CNP (Voxzogo^®^) in MPS IVA patients (NCT05845749). Voxzogo^®^ (vosoritide) is a stabilized analog of CNP that binds the natriuretic peptide receptor-B (NPR-B) on growth plate chondrocytes, increasing cGMP to antagonize FGFR3-MAPK signaling and thereby restore chondrocyte proliferation and differentiation [88]. This study, conducted at UCSF Children’s Hospital Oakland, involves administering daily subcutaneous injections of vosoritide at a dose of 15 µg/kg/day for 48 weeks to six pediatric patients aged 5–10 years. The trial also explores changes in linear and segmental growth, as well as biomarkers of bone metabolism. The use of CNP in combination with standard ERT could significantly improve skeletal manifestations. Establishing the dosing regimen will be critical as overexpression of CNP has shown side effects in MPS IVA mice [62].

### Antisense molecules: modulating aberrant splicing in the GALNS gene

Several deep-intronic variants lead to the activation of pseudogenes (PEs), affecting gene splicing and causing a shift in the mRNA reading frame by generating premature stop codons, which subsequently trigger nonsense-mediated mRNA decay [89]. In a study conducted by Bychkov et al. (2025), four PE-activating variants in the *GALNS* gene were identified in clinically diagnosed MPS IVA patients, resulting in the formation of frameshift and premature stop codons [90]. These variants were used to demonstrate the therapeutic potential of antisense oligonucleotides (ASOs) utilizing modified U7 small nuclear RNAs (modU7snRNAs) and circular RNAs (circRNAs) to correct aberrant splicing in HEK293 cells. Interestingly, the authors found that 20 bp modU7snRNA efficiently blocks all PEs, thereby restoring correct splicing [90]. When blocking PEs via circRNA, splicing restoration was observed with antisense sequences of 40 to 80 bp. Importantly, in one PE (c.1003-1570G > T), a 60-bp circRNA failed to recover splicing, potentially due to the formation of secondary structures [90]. Similar outcomes were reported by the authors when using 100 bp circRNAs, further supporting the premise that long circRNAs may adopt secondary structures [90]. In vitro and in vivo studies in MPS IVA models are still required to evaluate the feasibility of this innovative strategy.

## Future perspectives

**Fig. 3:**
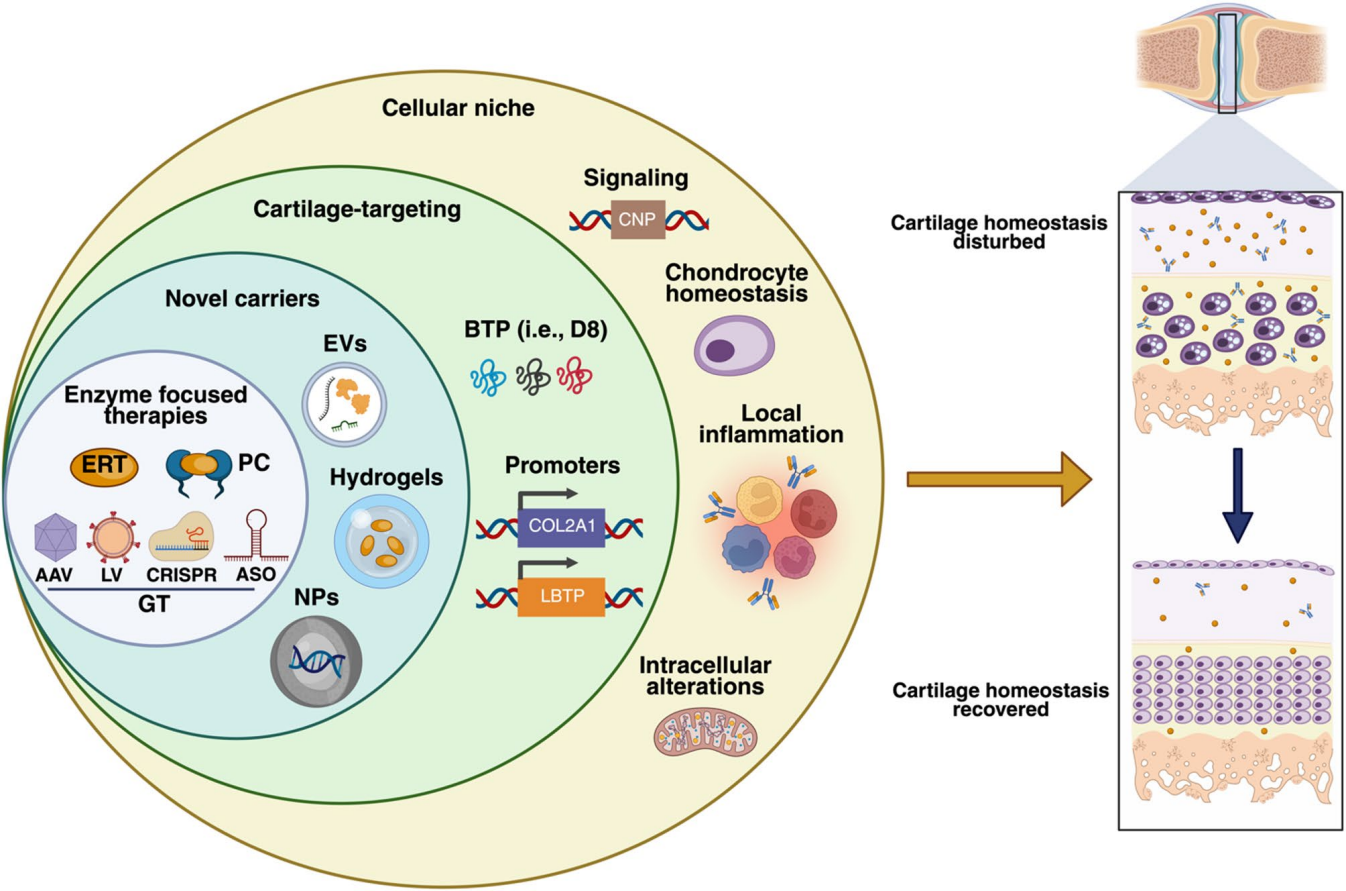
Current and new approaches in MPS IVA. Most therapeutic alternatives in MPS IVA focus on restoring GALNS activity through enzyme replacement therapy (ERT), pharmacological chaperones (PCs), and gene therapy (GT) using adeno-associated virus (AAV), lentivirus (LV), CRISPR/Cas9, and, more recently, antisense oligonucleotides (ASO). Novel carriers, such as nanoparticles (NPs), hydrogels, and extracellular vesicles (EVs), could act as potential enhancers of biodistribution by either their natural bone tropism or through engineering to increase bone and cartilage affinity through bone-targeting peptides (BTPs, i.e., D8). Likewise, the use of bone- and cartilage-specific promoters, including LMTP and COL2A1, driving GALNS expression, represents an innovative alternative to increase transgene expression in bone and cartilage. Additionally, recent evidence suggests that modulating chondrocytes through small molecules, such as C-type natriuretic peptide (CNP), may help alleviate cartilage pathology. Importantly, the well-known pro-inflammatory events, along with new insights into MPS IVA as a mitochondrial disturbance, pave the way for the inclusion of modulatory molecules. Undoubtedly, combinatory therapies addressing GALNS deficiency, along with modulation of the cellular niche, could significantly contribute to restoring cartilage homeostasis and thereby improving global MPS IVA pathology. This figure was created with Biorender.com

Substantial progress has been made in developing therapeutic alternatives for MPS IVA; however, several challenges, particularly the complete restoration of bone pathology, remain unmet. Promising strategies using bone-targeting promoters, tandem vectors, and bioengineered delivery systems, such as PEGylated hydrogels and extracellular vesicles (EVs) (Fig. 3), offer the potential to enhance biodistribution and bioavailability in various bone regions, including avascular zones.

GT remains one of the leading strategies in the pursuit of a curative approach in MPS IVA, with AAV-, LV-, and CRISPR/Cas9-based platforms yielding encouraging preclinical results. However, concerns about vector immunogenicity, off-target effects, and variable tissue tropism, particularly with AAV, require the development of optimized delivery platforms. Likewise, the exploration of combined strategies, including vectors expressing GALNS along with bone-modulating molecules, such as CNP, could improve skeletal manifestations by restoring the GALNS enzyme while modulating chondrocytes.

Importantly, innovative antisense-based approaches, such as modified U7 small nuclear RNAs (snRNAs) and circular RNAs, have opened a novel therapeutic avenue for correcting PE activation; however, their translation to in vivo and clinical practice requires further validation. Moreover, the identification of mitochondrial disturbances in MPS IVA chondrocytes [91], along with solid evidence of chronic inflammation [92–94], suggests that mito-inflammation may play a critical role in MPS IVA pathology. Nonetheless, no targeted anti-inflammatory or mitochondrial-directed strategies have yet been evaluated in preclinical MPS IVA models. Exploring immunomodulatory drugs or mitochondrial restoration (Fig. 3), in combination with GT or ERT, could help ameliorate secondary mechanisms and improve long-term outcomes.

Next-generation therapies should involve a combination of enzyme supply via ERT or GT, using cartilage-targeting strategies, along with modulation of the cellular niche (Fig. 3), to achieve the most substantial clinical benefits.

## Data Availability

No new data were generated during the preparation of this review. All information reported in this manuscript was extracted from publicly available scientific literature.

## Abbreviations

ASO: Antisense oligonucleotides
CAG: Cytomegalovirus Early Enhancer/Chicken β-Actin Promoter
CBh: Chicken β-Actin Hybrid Promoter fused to CMV enhancer
GT: Gene therapy
KS: Keratan sulfate
LBTP: Liver-Bone Tandem Promoter
LMTP: Liver-Muscle Tandem Promoter
LSPX: Liver-Specific Promoter based on the human alpha1-antitrypsin (hAAT)
MKC: MPS IVA knockout mouse
MSC-EVs: Mesenchymal stem cell-derived extracellular vesicles
MSCs: Mesenchymal stem cells
MTOL: MPS IVA knockout mouse tolerant to hGALNS
NVVs: Non-viral vectors
rhGALNS: Recombinant human GALNS
TBG: Thyroxine-Binding Globulin Promoter
VV: Viral vectors
WT: Wild-type
